# Femtosecond laser-assisted cataract surgery in a public teaching hospital setting

**DOI:** 10.1186/s12886-018-0693-6

**Published:** 2018-02-02

**Authors:** Alfonso Vasquez-Perez, Andrew Simpson, Mayank A. Nanavaty

**Affiliations:** 1 Sussex Eye Hospital, Brighton & Sussex University Hospitals NHS Trust, Eastern Road, Brighton, BN2 5BF UK; 2 Brighton & Sussex Medical School, University of Sussex, Falmer, Brighton, BN1 9PX UK

**Keywords:** FLACS, Femtosecond laser cataract surgery, Phacoemulsification, Teaching hospital, Conventional cataract surgery

## Abstract

**Background:**

To evaluate the efficiency and practicality of femtosecond laser assisted cataract surgery (FLACS) in a public teaching hospital setting using a mobile FLACS system compared to conventional phacoemulsification cataract surgery (CPCS).

**Methods:**

Ninety eyes from 90 patients underwent either FLACS or CPCS (45 in each group). Cataracts were graded using the Lens Opacities Classification System III system. Outcome measures included total surgery duration, femtosecond laser treatment time, vacuum time (VT), total phacoemulsification time (TPT) and total phacoemulsification power (TPP).

**Results:**

No differences were observed in the preoperative mean cataract grades and co-morbidities. FLACS took longer than CPCS with a mean difference of 5.2 ± 4.5 min (range: 0–18.8 min). The average femtosecond laser treatment time was 4.3 ± 3.4 min (range: 1–15.5 min). The VT was 2.51 ± 0.45 min (range: 1.59–4.10 min). Although not significant, TPT in FLACS showed a trend towards improvement (mean 1.0 ± 0.6 s; range: 0.1–2.4 s) compared to CPCS (mean 1.2 ± 0.6 min; range: 0.5–2.5 min). Whereas, TPP was significantly less in FLACS (mean 17.9 ± 5.0%; range: 5–27%) compared to CPCS (mean 20.3 ± 4.1%; range: 12.0–28.7%)(*p* = 0.031).

**Conclusions:**

The mobile FLACS system housed in the same operating room increased the surgical duration by 5.2 min. The average VT was 2.51 min, which was lower in comparison to published experience using non-mobile FLACS systems.

## Background

As cataract removal and its treatment options continue to evolve, the necessity of new innovations to circumvent standard phacoemulsification continues to be questioned. Femtosecond laser assisted cataract surgery (FLACS) has been shown to offer numerous potential advantages including a customized size and centration of capsulorhexis, astigmatic incisions and lens fragmentation of white and brunescent cataracts [1–6]. It also requires less phacoemulsification power and time thereby diminishing corneal endothelial injury; however its superiority over conventional phacoemulsification cataract surgery (CPCS) is still under scientific scrutiny [7–9]. Besides its cost, logistical challenges which include longer operating times and additional operating area represent major drawbacks that make cataract surgeons reluctant to adopt FLACS [4, 10–12].

In a public teaching hospital where cost and efficiency remain at the forefront of funding decisions, FLACS has generally been considered untenable due to the significant time and logistical burdens. For instance, due to the substantial clinical footprint of a femtosecond laser, almost all available platforms require their own dedicated room which is expensive, particularly in city hospitals where space comes at a premium. Additionally, unlike other theatre equipment, most FLACS lasers are completely immobile which means patients must be shuttled between rooms to complete surgery which not only adds time and increases risk of infection but also creates an additional liability burden for a hospital in the event that a patient sustains an injury during the transfer [13].

The LDV Z8 femtosecond laser system is the only completely mobile system with a small clinical footprint that can be shuttled in and out of rooms eliminating the need to move the surgeon and patient [14]. As space, time and general efficiency are core components of successful workflow in the public hospital setting, we decided to investigate how the LDV Z8 laser would perform in the public care setting compared to traditional phacoemulsification cataract surgery (CPSC) in terms of surgical time and patient exposure to phaco energy. Publications on femtosecond lasers, which explore operating times of FLACS versus CPSC, have examined stationary platforms, which require more theatre space [15–18].

In this study we evaluate how the use of a mobile femtosecond laser platform shuttled to the required operating room changes the operating times compared with CPCS with surgeons with varying surgical experience. This study was not aimed at identifying inter-surgeon differences but rather exploring the impact of having mobile FLACS system on the surgical duration in real time during theatre lists in a public teaching hospital.

## Methods

This prospective study included 90 eyes from 90 patients; 45 eyes underwent mobile FLACS and 45 eyes underwent CPCS at the Sussex Eye Hospital, Brighton & Sussex University Hospitals NHS Trust, Brighton, United Kingdom. This study was approved by the Audit and Research department at the Sussex Eye Hospital, Brighton & Sussex University Hospitals NHS Trust and followed the tenets of the Declaration of Helsinki.

Patients needing only routine cataract surgery were included in this study. No exclusions were made on the basis of cataract density, age and the depth of the eye socket. Patients with subluxated and traumatic cataracts were excluded.

The mobile laser was acquired through a local company (Instinctive UK Ltd., United Kingdom), which brought the device to the facility on scheduled surgery days and removed it the same day. Participants were recruited for the study on the day they were scheduled to undergo surgery whereby on days when the mobile Ziemer Z8 LDV was present, all patients were invited to participate in the study and offered an option for FLACS or CPCS. Patients whose surgery date did not coincide with the day the laser was present were informed, about the study and invited to participate in the CPCS arm. Patients who declined to take part in the study were offered cataract surgery as per usual United Kingdom National Health Service (NHS) protocols.

**Fig. 1:**
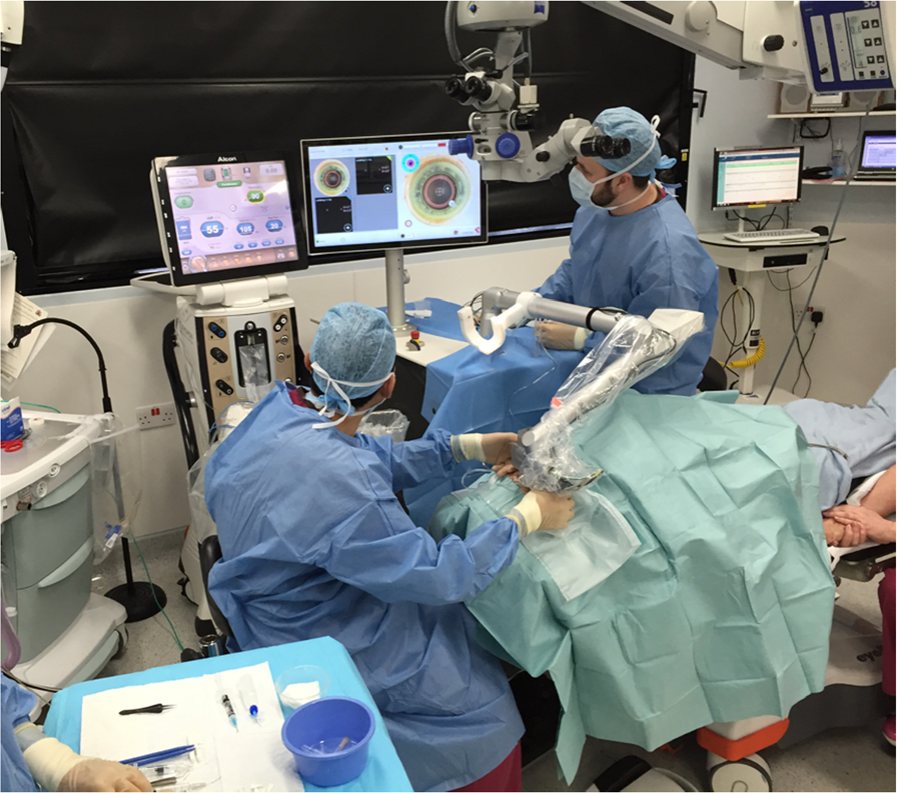
The arrangement of the mobile femtosecond laser, phacoemulsification machine, scrub trolley and the microscope. This shows the mobile femtosecond laser housed in the same theatre with the phacomachine next to the patient’s bed

Prior to the recruitment of patients in this study, each surgeon (a senior, mid and trainee level surgeon) was assessed on 10 consecutive FLACS cases and certified. Following this, 90 patients were consecutively recruited into the FLACS and CPCS groups between October 2015 and March 2016. All surgeries were completed without moving the patient and in the same operating room that contained a mobile femtosecond laser (Z8 LDV, Ziemer, Port, Switzerland) and the Centurion phacoemulsification platform (Alcon Surgical, Fort Worth, USA). The size of the operating theatre was 10 ft × 10 ft. The arrangement of the FLACS laser, phacoemulsification machine, scrub trolley and the microscope is shown in Fig. 1.

On the day of the surgery, patients were dilated with G. Tropicamide 1% (Minims, Bausch & Lomb UK limited, UK) and G. Phenylephrine 2.5% (Minims, Bausch & Lomb UK limited, UK). Cataract density was graded using LOCS III grading [19]. At the time of the surgery, an independent theatre practitioner recorded the ‘total surgery duration’ or time from betadine 5% (Minims, Bausch & Lomb UK limited, UK) application to speculum removal at surgery completion.. Additionally for FLACS cases, time was recorded starting when the suction ring touched the eyeball before the suction was applied and the ring was removed to after the suction was released. This time was labeled as ‘Femtosecond laser treatment time’ and included anything that happened between the above two time points such as time lost due to docking issues, laser planning delays, re-checking the laser parameters whilst the ring still touching the eye but prior to vacuum application, re-dockings due to failed suction, etc. The duration of applied vacuum via the suction ring for the treatment was recorded as ‘vacuum time’ (VT). Any intraoperative complication or event was also noted at the end of the procedure. Total phacoemulsification time (TPT) and total phacoemulsification power (TPP) was recorded from the phacoemulsification machine.

The primary outcome measure was total surgical duration. Secondary outcome measures included femtosecond laser treatment time, VT, TPT and TPP.

### Surgical technique

All surgeries were performed under topical anaesthesia (G. Proxymethacaine Minims, Bausch & Lomb UK limited, UK)). In FLACS cases, a disposable suction ring was applied to the eye and was filled with balance salt solution creating a fluid-filled interface. The mobile arm of the laser system was then docked over the cornea. Once the integrated OCT imaged the ocular structures and the surgeon confirmed the parameters, the laser was applied starting with lens fragmentation in four quadrants and then capsulotomy with a predetermined diameter. Laser power was graded as per the density of the cataract based on LOCS III cataract grading in that the energy was titrated based on the surgeons experience of the laser with the grade of the cataract. If the docking was not successful in the first attempt, subsequent attempts were made until a successful docking was achieved. The time of when the suction ring was removed was noted. All patients, irrespective of FLACS or CPCS, received manual superior corneal incisions and 2 paracentesis at 0 and 180 degrees. In the FLACS cases, the free-floating anterior capsulorhexis flap was removed with capsulorhexis forceps after viscoelastic injection followed by hydrodisection. As the nucleus was already fragmented the nucleus was split using primary chop technique. Following the removal of nucleus, a bimanual irrigation and aspiration was performed and a single piece hydrophilic acrylic intraocular lens was implanted into the capsular bag. Intracameral Cefuroxime (Aprokam, Thea Pharmaceuticals, France) was injected after thorough irrigation and aspiration to remove the residual viscoelastic. The paracentesis wound were hydrated and the speculum was removed. The time of the speculum removal was noted in all cases.

### Statistical analysis

All data was recorded on Microsoft Office Excel® 2010 (Microsoft® Corporation, USA). Normality of all the data was tested by Kolmogorov-Smirnov test. The SPSS statistics version 22.0 (International Business Machines® Corporation) was used for all statistical analysis. TDS, TPT and TPP between two groups were compared using a 2 sample unpaired 2-tailed t test with pooled variance. Differences with a *P* value less than 0.05 were considered statistically significant.

## Results

There were no significant differences in age and sex between the two groups. The mean age of the patients was 72 ± 10.4 (range: 61–83 years). In the FLACS group there were 20 females and 25 males whereas in the CPCS group there were 27 females and 18 males. In the FLACS group, 23 patients were Caucasians and 2 were Afro-Caribbean. Whereas, in the CPCS group, 44 patients were Caucasians and 1 was Afro-Caribbean and 1 was Chinese. There was no significant difference in the LOCS III grading of the cataracts between the FLACS and CPCS groups. No intraoperative complications were reported in either of the groups.

**Fig. 2:**
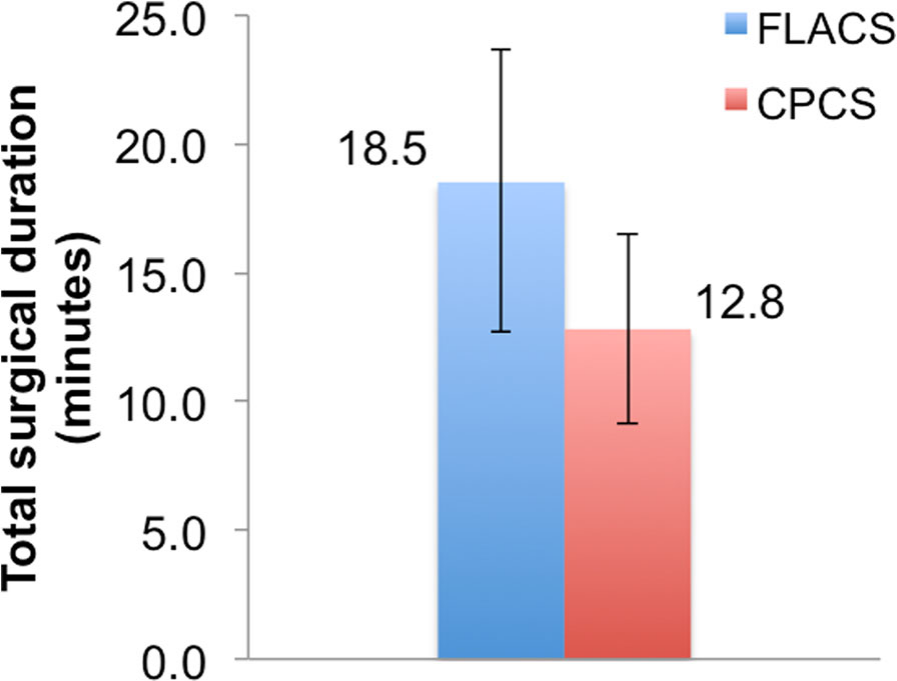
Graph showing difference in total surgical duration between FLACS and CPCS. FLACS showed significantly longer total surgical duration

**Fig. 3:**
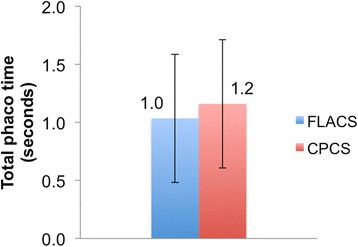
Graph showing difference in TPT (seconds) between FLACS and CPCS. FLACS showed less TPT but it was not statistically significant

**Fig. 4:**
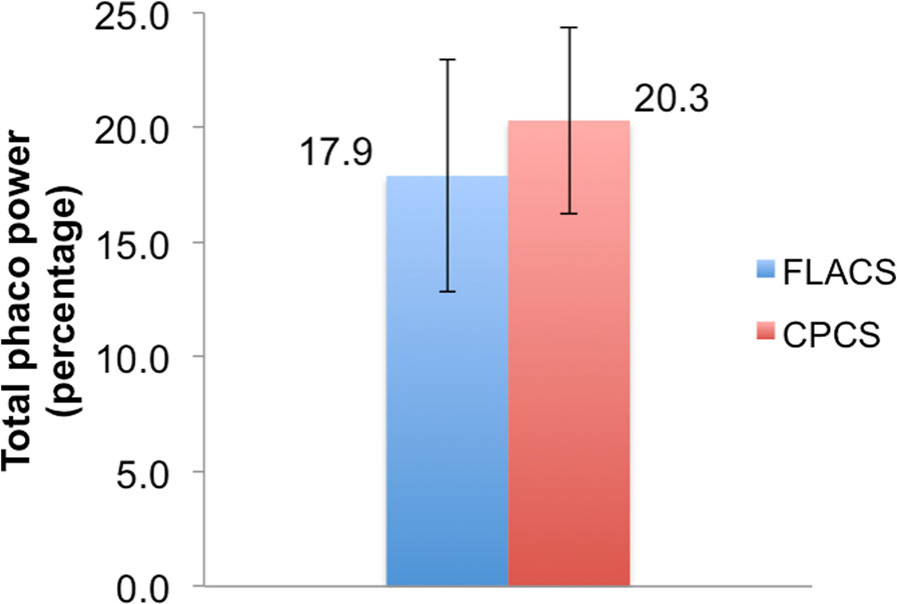
Graph showing difference in TPP (percentage) between FLACS and CPCS. This was significantly less with FLACS

As shown in Fig. 2, FLACS (mean: 18.5 ± 5.1 min; range: 12–32.4 min) took significantly longer compared to CPCS (mean 12.8 ± 3.7 min; range: 4.5–23.2 min) (*p* < 0.0001). The mean of the difference in the total surgical duration between FLACS and CPCS was 5.2 ± 4.5 min (range: 0–18.8 min). The average femtosecond laser treatment time was 4.3 ± 3.4 min (range: 1–15.5 min). The VT was 2.51 ± 0.45 min (range: 1.59–4.10 min). As shown in Fig. 3, although TPT was less in FLACS (mean 1.0 ± 0.6 s; range: 0.1–2.4 s) compared to CPCS (mean 1.2 ± 0.6 min; range: 0.5–2.5 min) it was not statistically significant (*p* = 0.348). Whereas, TPP was significantly less in FLACS (mean 17.9 ± 5.0%; range: 5–27%) compared to CPCS (mean 20.3 ± 4.1%; range: 12.0–28.7%)(*p* = 0.031) (Fig. 4). Although not objectively assessed but the learning curve of FLACS docking skills were similar amongst all grades of the surgeons. For the less experienced surgeons acquiring Femtosecond laser cataract surgery skills improved their overall confidence. Total surgery time for less experienced surgeons was longer for both CPCS and FLACS and shorter for more experienced surgeons.

## Discussion

FLACS has proven to be an effective and safe technique [1, 7–9, 11, 12, 14] but comparing it with conventional phacoemulsification cataract surgery, FLACS demands logistical considerations which can be challenging to overcome in the public teaching hospital setting where maintaining efficiency is critical. Additional operating times, extra operating space and the consideration for the patients/surgeon to transfer from one room to another to complete the procedure have all been associated with FLACS [4, 10–12, 15]. Information can be found in peer reviewed literature about the potential advantages and outcomes of FLACS [4, 15–18, 20], however, little has been published on how the use of mobile FLACS system affects the amount of operating time in real life in comparison to CPCS.

From the time of betadine application to the point of removal of the speculum, we found that cases undergoing FLACS spent an average of five minutes more compared with CPSC. This difference did not dissipate with surgical experience. In a non-comparative study by a single surgeon using mobile Ziemer LDV Z8, the TDS was reported to be 16.3 ± 4.5 min going down to 12.5 ± 1.1 min once the learning curve had been reached by an experienced surgeon [21]. Two retrospective studies on static FLACS platforms housed in the same operating room (Catalys Precision Laser System, Abbot medical optics, Santa Ana, CA), evaluated surgical times in FLACS compared to CPCS. Lubahn et al. [17] in 162 cases, considered “total operative time”, as the time the patient entered the operating room to the patient leaving the room. Grewal et al. [16] in their study in 166 cases, among different parameters, recorded “procedure time” as the time from the suction ring application in FLACS or corneal incisions in CPCS to the speculum removal. This last parameter in Grewal’s study [16] was similar to our primary outcome measure (total surgical duration). Lubahn [17] reported a mean difference of 14 min longer for FLACS whilst Grewal [16] reported only 9 min. In another study but Bali et al. [15] comparing FLACS with CPCS, a non-mobile FLACS system was housed outside the operating room and the mean operating theatre time was 15.7 min for CPCS and 19.8 min for FLACS.

Because different studies have reported FLACS times differently, we decided to report the actual VT and femtosecond laser treatment time. Femtosecond laser treatment time would include VT and any additional time taken before and after suction was switched on and off. This included time to position the suction ring on the cornea, re-checking the laser parameters for the final time with the suction ring in place but before application of suction, any loss of suction and re-docking attempts, etc. A few results of VT have been published with various non-mobile platforms. Grewal et al. [16] in their study using Catalys platform, analyzing the impact of learning curve on the time durations in FLACS, noted a VT of 3.35 min after the surgeon gained experience on few cases. Whereas, Chang et al. [20], using LensAR® platform, reported the VT of 6.72 ± 4.57 min (range: 2–28 min). Using Victus® platform, Baig et al. [21] reported VT of 3.6 ± 0.25 min. Rivera et al. [22] in their study reported VT of 3.75 min (range: 2.5–9.38 min) with Catalys® and 2.88 min (range: 1.13–4.31 min) with LenSx®. Kerr et al. [23] reports the shortest time 3.05 min using the Catalys® system. In comparison to above studies, we found a lower VT of 2.51 ± 0.45 min (range: 1.59–4.10 min) with the mobile Femto LDV Z8.

Although the findings of Grewal et al. [16] and Lubahn et al. [17] cannot be compared directly due to differences in the study outcome measures, our results showed lower total surgical duration with a mobile FLACS system. But like their studies [16, 17], we also found a significantly longer total time for FLACS compared to CPCS which was consistent across surgeons’ grades. As femtosecond laser treatment time reports have been similar across different laser platforms; [16–18, 20, 21, 23] the longer operating times of both studies [16, 17] compared to our results undoubtedly may be due to logistical and workflow differences. With non-mobile platforms, even if the femtosecond laser is housed in the same operating room the patients need to be transferred from the laser bed to the microscope, painted and draped before continuing with phacoemulsification under a different operating microscope. Unique to our study is that using a mobile laser platform with varying grades of the surgeon, neither the patient nor the surgeon needed to move irrespective of the patient undergoing FLACS or CPCS. We did not analyze the inter surgeon difference as the aim of our study was only to evaluate the real time difference between the procedures in a teaching environment.

We found statistically significant less TPT and TPP used in the FLACS group but these findings are well known advantages of femtosecond laser reported in vast number of publications [1, 2, 4, 7–12].

Using the only available mobile laser platform (Ziemer LDV, Z8) we found the shorter difference in operating times among published comparative studies of FLACS. This laser uses a fluid-filled patient interface and has an optical coherence tomography (OCT) integrated directly into the hand piece that uses the same optics as the laser beam [18]. In our experience, we found that the most important aspects of the Z8 system were its mobility, which simplifies patient flow, and its size, requiring only a small extra space in the operating room. In a public healthcare setting, both of these aspects are beneficial as caseload and room space are often limiting. Although we did not test other modules on the Z8, it can also be used for refractive surgery and keratoplasty which could be beneficial when sharing the device among other departments in a comprehensive ophthalmic teaching center. Moreover, studies have shown pupil miosis after femtosecond laser application during FLACS [24, 25]. However, in our study, we did not use any preoperative non-steroidal anti-inflammatory eye drops and still did not find significant pupil miosis. We believe this is because previous studies report pupil miosis after femtosecond laser application were performed using non-mobile laser systems housed in a different room and there was a small time delay between laser application and phacoemulsification. Whereas in our study, the mobile Z8 FLACS system was housed in the same operating room and the femtosecond laser application was commenced after painting and draping the patient for the cataract surgery (effectively causing now delay between femtosecond laser application and phacoemulsification).

In terms of safety and efficiency of FLACS in the public hospital setting, intra operative patient transfer has not been addressed appropriately in any study evaluating FLACS; despite requiring additional transport time and staff, it could potentially introduce risks of infections and secondary injuries when patients are moved to a different room. There are hygienic and ethical issues with patient’s shuttle between non-mobile laser not housed in the same room and the operating room [23]. And if the patient requires hook or iris expanders before the use of non-mobile femtosecond laser in a clean room outside the operating room then this could be an issue from the point of view of hygiene and infection.

Limitations of our study include the relatively low number of cases and that all three surgeons despite vast experience in CPSC had only limited experience in FLACS. We could assume that with additional experience there might be a reduction in operating times as already shown by a previous study [16]. However we believe that FLACS is a safe and a well-developed technique and has a fast learning curve compared with CPSC. This study also did not focus on the astigmatic correction with femtosecond laser based incision and all surgeries where planned with superior corneal incisions.

## Conclusions

Public hospitals must consider the cost/benefit and understand in which patient cases FLACS can become truly beneficial [26–29]. as well as how to share a device across multiple sub-specialties within the ophthalmology department. Centers willing to offer patients the advantages of this new technology in addition to the direct cost related with the laser, must budget the running cost of an operating room and make an effective strategy to reduce operating times and not significantly decrease their caseload. Mobile FLACS system apart from the proven advantages of FLACS requires less space and also appears to perform better in operating times than stationary platforms, however further evaluation of these parameters with direct clinical comparison of mobile and non-mobile FLACS systems are needed.

## Abbreviations

CPCS: Conventional phacoemulsification cataract surgery
FLACS: Femtosecond laser assisted cataract surgery
TPP: Total phacoemulsification power
TPT: Total phacoemulsification time
VT: Vacuum time
